# Protection of the Photosynthetic Apparatus from Extreme Dehydration and Oxidative Stress in Seedlings of Transgenic Tobacco

**DOI:** 10.1371/journal.pone.0051443

**Published:** 2012-12-05

**Authors:** Concepción Almoguera, Pilar Prieto-Dapena, José-María Personat, Javier Tejedor-Cano, Marika Lindahl, Antonio Diaz-Espejo, Juan Jordano

**Affiliations:** 1 Instituto de Recursos Naturales y Agrobiología de Sevilla, Consejo Superior de Investigaciones Científicas (CSIC), Seville, Spain; 2 Instituto de Biología Vegetal y Fotosíntesis, Consejo Superior de Investigaciones Científicas (CSIC), Seville, Spain; Instituto de Biología Molecular y Celular de Plantas, Spain

## Abstract

A genetic program that in sunflower seeds is activated by Heat Shock transcription Factor A9 (HaHSFA9) has been analyzed in transgenic tobacco seedlings. The ectopic overexpression of the HSFA9 program protected photosynthetic membranes, which resisted extreme dehydration and oxidative stress conditions. In contrast, heat acclimation of seedlings induced thermotolerance but not resistance to the harsh stress conditions employed. The HSFA9 program was found to include the expression of plastidial small Heat Shock Proteins that accumulate only at lower abundance in heat-stressed vegetative organs. Photosystem II (PSII) maximum quantum yield was higher for transgenic seedlings than for non-transgenic seedlings, after either stress treatment. Furthermore, protection of both PSII and Photosystem I (PSI) membrane protein complexes was observed in the transgenic seedlings, leading to their survival after the stress treatments. It was also shown that the plastidial D1 protein, a labile component of the PSII reaction center, and the PSI core protein PsaB were shielded from oxidative damage and degradation. We infer that natural expression of the HSFA9 program during embryogenesis may protect seed pro-plastids from developmental desiccation.

## Introduction

In plant seeds the developing zygotic embryo survives desiccation. Mature seeds can germinate after prolonged storage leading to seed aging. The existence of different genetic programs that regulate at the same time seed longevity and embryonic desiccation tolerance has been proposed [1]–[4]; one of such programs is under control of Heat Shock transcription Factors (HSFs). HaHSFA9 in sunflower (*Helianthus annuus*, L.), and the orthologous HSFs (HSFA9) are expressed specifically in developing seeds [5], [6]. The known target genes activated by HSFA9 (the HSFA9 program) encode different Heat Shock Proteins (HSPs), among them HSP101 and distinct subsets of small Heat Shock Proteins (sHSPs) that belong to the cytosol-localized CI and CII classes [1]–[3], [6]. In sunflower these sHSPs include polypeptides that accumulate specifically (or predominantly) in developing seeds at normal growth temperature, but they do not so (or do it to lesser extent) in response to heat stress in vegetative organs. The seed-specific, or the 35S-driven overexpression of HaHSFA9 resulted in enhanced accumulation of the same subsets of cytosolic sHSPs [1], [2]. In parallel, we observed enhanced seed longevity [1] and achieved tolerance to drastic levels of dehydration in vegetative organs [2], respectively.

The results of ectopic overexpression of the HSFA9 program in transgenic tobacco (*Nicotiana tabacum* L., [2]) turned our attention to the green organs, which survived drastic dehydration better than roots. The tolerated dehydration -losses of ≈98% of the initial amount of water- implied reaching very low water potentials (e.g., Ψ below −30 MPa). Survival under these conditions indicated the protection of membranes, including plastidial membranes. These observations lead us first to investigate if the activation of the HSFA9 program involves plastidial sHSPs. These sHSPs are encoded by the nuclear genome and targeted to the chloroplast. Precedents in the literature have showed that the plastidial sHSPs of higher plants can protect photosynthetic membranes and their components (e.g., [7]–[9]). However, the previous *in vivo* studies have investigated resistance to damage caused only by very moderate levels of stress, e.g., heat stress, high-light stress or both combined.

In chloroplasts, PSII is a critical site of damage by stress conditions such as dehydration, heat, high-light, and UV-B light [10]. PSI has generally been regarded as a stress-resistant photosystem compared to PSII, at least in normal plants subjected to moderate stress levels. Damage to PSI has been considered to take place only rarely *in vivo,* as in certain plant species or under specific environmental conditions, such as chilling [11]. In particular, desiccation in the dark would irreversibly damage PSI of normal plants, but not of resurrection (desiccation-tolerant) plants (e.g., [12], [13]). Reactive oxygen species (ROS), such as superoxide radicals, hydroxyl radicals and hydrogen peroxide (H2O2), are produced in the stressed chloroplasts. These ROS cause critical oxidation and damage of PSII. Among the intrinsic components of the reaction center of PSII, the chloroplast-encoded D1 protein (PsbA) is particularly vulnerable to ROS damage [14], [15]. The damage of the D1 protein causes reduction of oxygen evolution in the PSII and disruption of the photosynthetic electron flow [10]. Therefore, the D1 protein would be among the candidate targets that could be shielded from oxidative damage under the drastic stress conditions analyzed *in vivo* in this report. It should be remarked that these stress conditions were strong enough to damage not only PSII but also PSI within chloroplast of control non-transgenic plants.

In this work, it was found that the HSFA9 program includes plastidial sHSPs that are normally expressed in developing seeds but are present only in lower abundance in heat-stressed leaves. The ectopic overexpression of HaHSFA9 in green organs of tobacco seedlings protected membrane-protein complexes of the two photosystems. It also shielded the D1 protein of PSII and its synthesis from oxidative damage inferred by drastic dehydration or by very harsh treatments with H_2_O_2_ (e.g., exposure to up to 200 mM H_2_O_2_ for 24 h in the dark). The PSI core protein PsaB was also protected. Thus, protection at different structural levels might explain the limited and recoverable damage of PSI and PSII observed in the 35S:A9 seedlings. In contrast, heat acclimation of control non-transgenic seedlings induced thermotolerance but not resistance to the drastic stress conditions used in this study. Thus, target genes not activated in a typical heat-acclimation response in vegetative organs, but included in the HSFA9 program must explain the unusual stress resistance of the 35S:A9 seedlings. Among them, those encoding the cytosolic and plastidial seed sHSPs may have a partial role. We propose that the normal expression of the HSFA9 program, which occurs during zygotic embryogenesis, would protect maturing seed plastids from desiccation. The results reported here might open new ways to engineering the stress tolerance of photosynthetic organs.

## Results

### The seed HSFA9 program includes plastidial sHSPs that ectopically accumulate at high levels in the 35S:A9 seedlings

**Figure 1:**
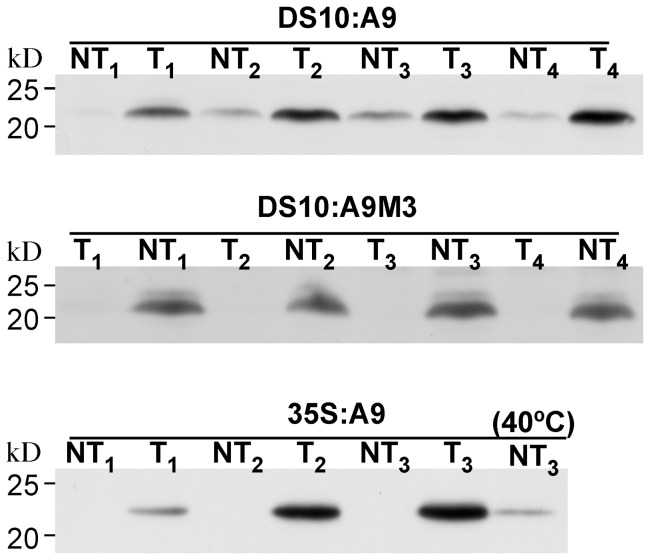
Effects of gain and loss of function of HaHSFA9 on the accumulation of plastidial sHSPs in seeds and seedlings. Immunoblot detection with the anti-HSP21 antibody at 1/2,500 dilution. Different transgenic lines (T) were compared with sibling non-transgenic (NT) lines. From top to bottom: DS10:A9 seeds, DS10:A9M3 seeds, and 35S:A9 seedlings. Protein amounts loaded: 25 μg total protein for the seed samples and for the 40°C seedling sample; 15 μg for the rest of seedling samples. Enhanced detection conditions were used for the DS10:A9M3 samples with respect to DS10:A9 (90 min instead of 5 min exposure, compare the respective NT signals). The high-level accumulation of plastidial sHSPs in unstressed seedlings of the 35S:A9 lines was compared with that in NT seedlings after heat-acclimation for 3 h at 40°C (40°C). Molecular mass markers (kD) are indicated to the left.

We first investigated whether genes encoding plastidial sHSPs are activated in the HSFA9 program. To this end, commercial antibodies raised against HSP21 -a chloroplast-localized sHSP from Arabidopsis- were used. The accumulation of polypeptides detected by these antibodies was determined in transgenic lines that up-regulate (DS10:A9) or down-regulate (DS10:A9M3) the HSFA9 program in seed [1], [3]. The observed patterns were then compared with the accumulation of these polypeptides in vegetative organs of heat-acclimated non-transgenic lines, and in non-stressed 35S:A9 lines. Each transgenic line (T) was compared with its sibling non-transgenic (NT) control syngenic line obtained by Mendelian segregation. The T lines represent different single integration events of the respective *HaHSFA9* transgenes in homozygosis. Different sibling T/NT pair lines were used for the experiments described in Figure 1 (see the legend). The same three pairs of sibling 35S:A9 lines [2] were also used for the rest of the experiments in this report. Mature seeds of the DS10:A9 lines, which display gain of function of the HSFA9 program, showed consistently higher accumulation levels of HSP21-like polypeptides compared to the NT control lines (Figure 1, top). Conversely, loss of function of the HSFA9 program in the seeds of DS10:A9M3 lines abolished accumulation of these polypeptides compared to the respective NT control lines (Figure 1, middle). The specificity of the anti-HSP21 antibodies is evident, as the molecular mass of the detected polypeptides is consistent with that for processed plastidial sHSPs (mature forms without the chloroplast transit peptide). Furthermore, we did not detect the smaller polypeptides recognized by anti-CI- and anti-CII-sHSP antibodies: compare Figure 1 with data in our previous publications [1]–[3]. Thus, genes encoding plastidial sHSPs are activated during seed maturation as part of the HSFA9 program, as previously shown for different sHSPs. In control (non-stressed) NT seedlings we could not detect accumulation of HSP21-like polypeptides; in contrast seedlings of the 35S:A9 (T) lines accumulated these plastidial sHSPs. The accumulation level of plastidial sHSPs in the 35S:A9 seedlings was consistently much higher than observed with heat-acclimated NT seedlings, where treatments for 3 h at 40°C induced plastidial sHSPs as expected (Figure 1, bottom). We also showed partial association to thylakoid membranes, under unstressed growth conditions, of the plastidial sHSPs overexpressed in the T lines. These plastidial sHSPs showed a thylakoid-membrane association at least as strong as that of the PsbP protein of PSII (Figure S1).

### Protection of PSII and PSI against severe dehydration and oxidative stress in the 35S:A9 seedlings

**Figure 2:**
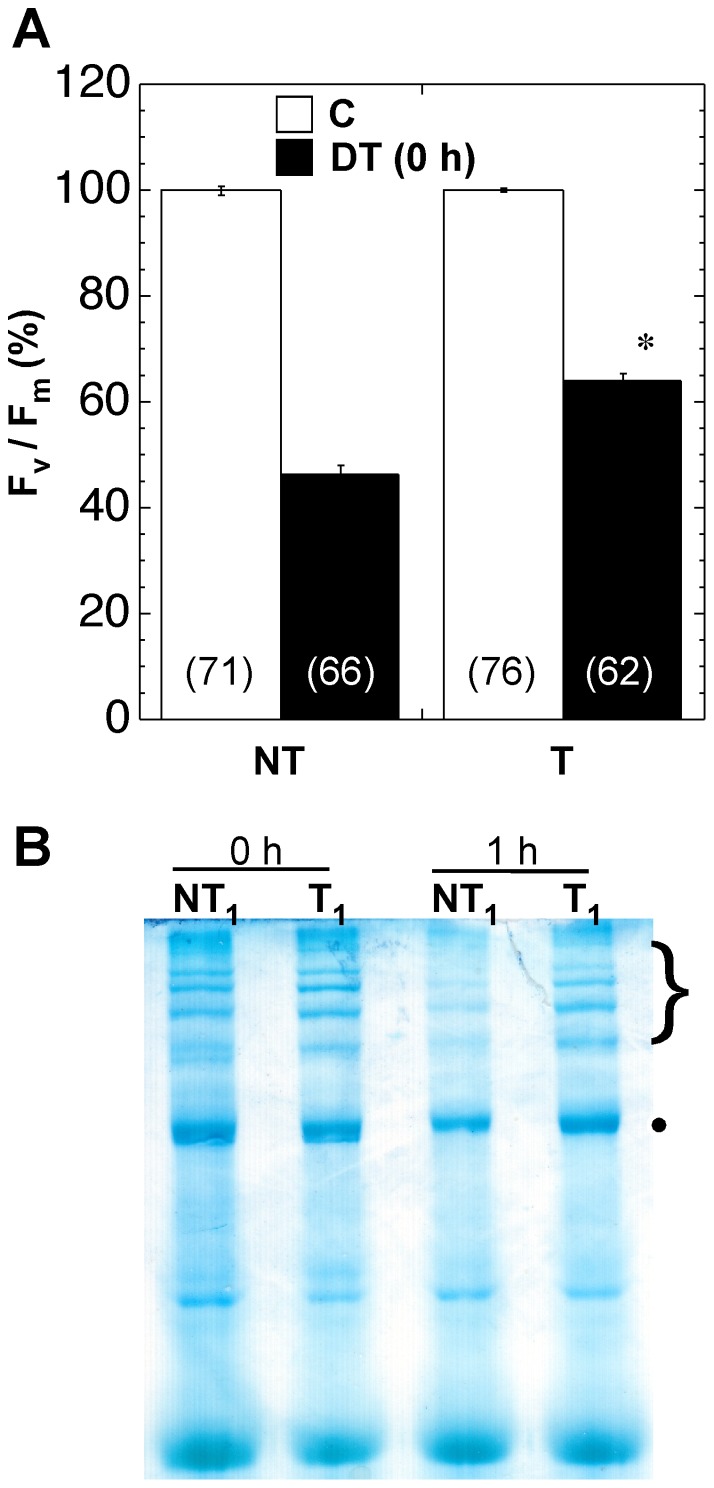
Protection of PSII from damage caused by severe dehydration. (**A**) Comparison of maximum quantum yield (F_v_/F_m_) values of transgenic seedlings (T) with those for the non-transgenic seedlings (NT), determined in control growth conditions (C) and immediately after the dehydration treatment [DT (0 h)]. Average values obtained in 4 to 5 independent experiments performed with two different T/NT line pairs. The difference observed for the DT (0 h) values was statistically significant (F = 194.41, P = 0.0001) as indicated by the asterisk. Numbers in brackets indicate the different F_v_/F_m_ determinations performed in each case. (**B**) Enhanced stability of PSII membrane protein complexes. The complexes were visualized by BN-PAGE using samples prepared from dehydrated seedlings analyzed immediately after the dehydration treatment (0 h), and following rehydration for 1 h (1 h). The gel was stained with colloidal Coomassie blue. Symbols (•, and the bracket on top) mark the dimeric PSII complex and the PSII super-complexes mentioned in the text. Representative results for the T_1_/NT_1_ sibling pair are shown.

**Figure 3:**
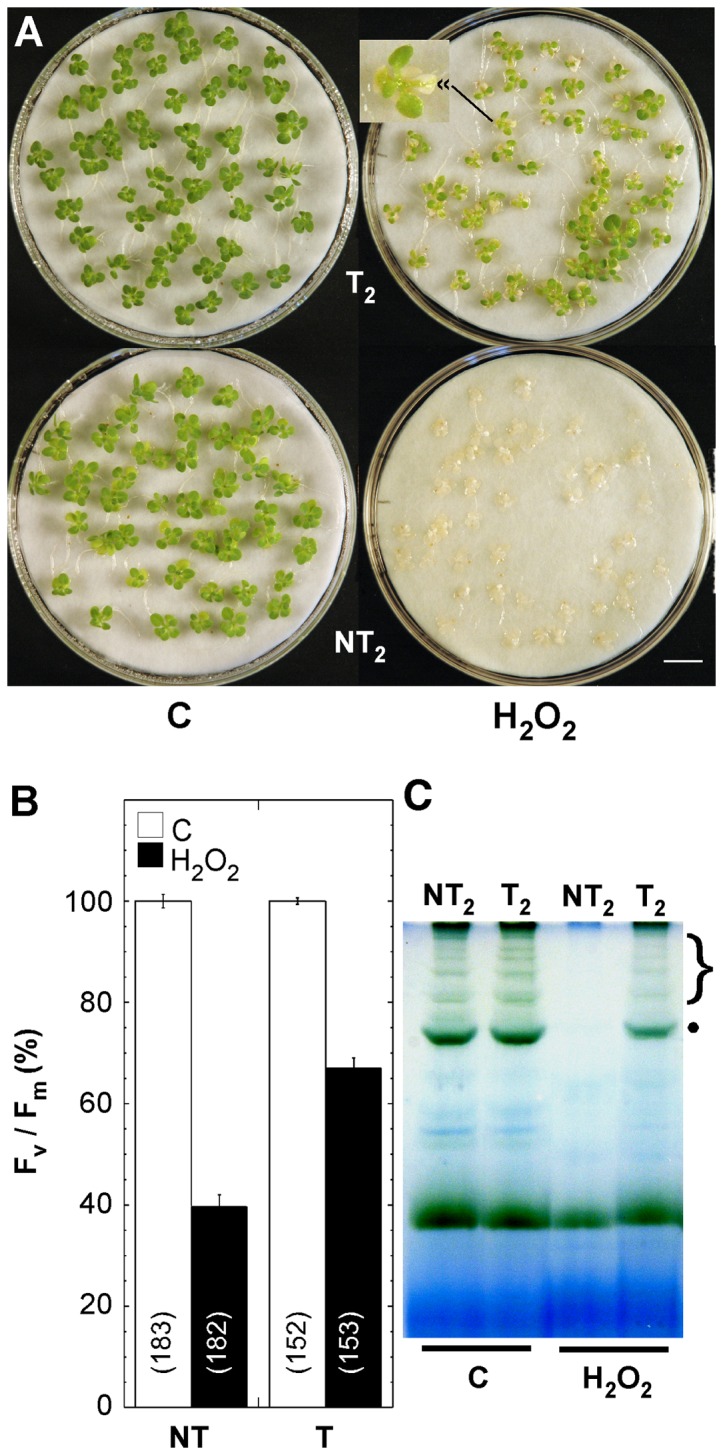
Protection of PSII from damage caused by drastic oxidative stress conditions. (**A**) The 35S:A9 seedlings survive drastic oxidative stress conditions. Representative results for the T_2_/NT_2_ line pair are shown. Differences in green color were evident between seedlings grown in control conditions (C). Most 35S:A9 seedlings (T_2_) survived treatments for 24 h with 200 mM H_2_O_2_ (H_2_O_2_) that caused complete death of sibling NT_2_ seedlings. The 3x-magnified inset shows bleaching of the cotyledons in the surviving T_2_ seedlings. Scale bar, 1 cm. (**B**) Comparison of maximum quantum yield (F_v_/F_m_) of PSII between 35S:A9 (T) and NT seedlings after control (C) and H_2_O_2_ treatments (H_2_O_2_). Average values for nine independent experiments performed with three different T/NT line pairs. Statistical difference between the F_v_/F_m_ values (F = 265.58, P = 0.0001) and rest of symbols indicated as in Figure 2A. (**C**) PSII membrane protein complexes of 35S:A9 seedlings partially resisted the H_2_O_2_ treatments. Thylakoids from control (C) and H_2_O_2_ treated seedlings (H_2_O_2_) were analyzed essentially as indicated in Figure 2B. The H_2_O_2_ samples were prepared immediately after the treatment and the gel was photographed after BN-PAGE without further staining. Symbols for PSII complexes as in Figure 2B.

We previously showed that 3-week-old 35S:A9 (T) seedlings survive drastic dehydration; whereas 100% NT siblings die, ≈80% of the T seedlings recover from dehydration treatments (DT) up to Ψ ≈−20 MPa; the root is lost and only leaves and green tissue of the T seedlings survive to different extents after DT up to Ψ ≈−40/−50 MPa. Seedling survival was optimized by performing DT in the dark [2]. Here, the described DT2 protocol [2] was modified to allow performing DT using seedlings placed on glass microscope slide during the stress treatment (see “Material and Methods”). Severe dehydration was thus achieved to contents of 0.5–1.0 g water per gram of dry weight (corresponding to Ψ≈−20 MPa and lower), enabling measurement of F_v_/F_m_ of PSII immediately after DT (see “Materials and Methods” for details). Figure 2A shows that the maximal efficiency of PSII declined after DT, albeit to levels with respect to the control treatment values that were significantly lower for the NT seedlings compared to T seedlings. These results indicate protection of PSII in seedlings of the 35S:A9 plants. Damage after severe dehydration involves the dismantling of membranes. Dehydration damage also leads to the oxidation of cellular components by ROS (see [16] and references therein). Therefore, we also investigated whether the 35S:A9 seedlings can survive treatments with H_2_O_2_ in the dark, and if the PSII is indeed protected from oxidative damage. In initial experiments we found that the PSII of T seedlings was protected to different extent after treatments for 24 h with 50 to 500 mM H_2_O_2_. Treatment with 200 mM H_2_O_2_ was selected as the standard experimental condition, because it was the highest H_2_O_2_ concentration that allowed survival of ≈100% of the T seedlings. The photosynthetic organs –except cotyledons- conserved their green color. In contrast, the NT sibling bleached and did not survive treatments with 200 mM H_2_O_2_ (see representative results in Figure 3A). In Figure 3B, we also show that immediately after the 200 mM H_2_O_2_ treatments, the F_v_/F_m_ values declined to levels with respect to the control treatment values that were significantly lower for the NT than for the T seedlings. The results of Figures 2 and 3 demonstrate protection of vegetative organs and PSII integrity in the 35S:A9 plants.

Thylakoid chlorophyll-protein complexes were also analyzed using Blue-Native gels. These complexes were separated after mild solubilization using β-dodecyl-n-maltoside. We could detect -for example- monomeric PSII, dimeric PSII, and super-complexes with dimeric PSII associated to light-harvesting proteins. These complexes (Figure 2B), which in this gel system are not separated from PSI complexes, were assigned based on comparison of their mobility with those of thylakoid chlorophyll-protein complexes resolved by BN-PAGE reported in the literature (see e.g., [17]–[19]). Under control conditions, the detected complexes were similar for the NT and T seedlings (Figure 3C). Both the dehydration and H_2_O_2_ treatments destroyed the plastid membrane complexes in the NT seedlings (Figures 2B and 3C). In contrast, some complexes resisted the stress treatments in the T seedlings. This stress-protection was perhaps more evident for PSII-containing complexes. Notably the protection of PSII super-complexes, which are regarded as the functional photosynthetic units, was higher (Figures 2B and 3C). Interestingly, immediately after DT all complexes apparently resisted dehydration and appeared similar in both NT and T seedlings (Figure 2B, DT samples 0 h). However, following rehydration for only 1 h after DT most NT complexes disappeared, whereas T seedling complexes resisted (Figure 2B, DT samples 1 h). These results contrast with what observed for the H_2_O_2_ treatments, where protection of the complexes in the T seedlings could be observed immediately after treatment (Figure 3C). Most likely the rapid and drastic dehydration conditions used for DT do not allow the immediate disassembly and degradation of the complexes in the NT seedlings. Indeed, we reported that upon DT2 the water content is reduced below 2.5 g water/g dry weight after only 3 h dehydration [2]. Thus, at least the complexes of PSII in thylakoid membranes of 35S:A9 plants are indeed protected against the oxidative damage imposed by the severe DT and H_2_O_2_ treatments.

**Figure 4:**
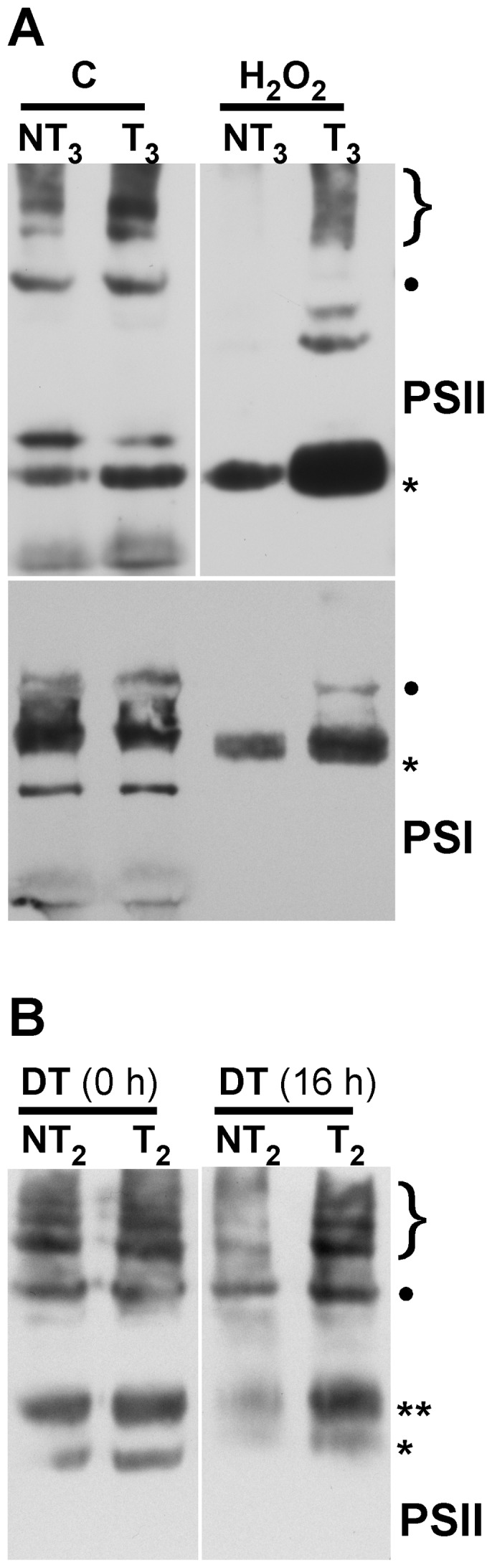
Membrane protein complexes of PSII and PSI survive in the 35S:A9 seedlings. (**A**) Western detection of complexes after the H_2_O_2_ treatments. The PSII complexes (top) separated by BN-PAGE were detected using anti-D1 (DE-loop) antibodies at 1/5,000 dilution. The PSI complexes (bottom) were detected using anti-PsaB antibodies at 1/4,000 dilution. PSII symbols: •, and the bracket on top respectively mark the dimeric PSII complex, and the PSII-LHCII super-complexes. The asterisk marks the CVII (CP43-less PSII monomer) complex. PSI symbols: • marks the PSI-LHCI super-complex that co-migrates in our gel system with the dimeric PSII complex; the asterisk marks the PSI monomer. (**B**) The PSII complexes also withstand drastic dehydration. The thylakoid samples were analyzed immediately (0 h) after the dehydration treatment (DT), and following rehydration for 16 h, DT (16 h). In this case the complexes were detected using anti-D1 (C-terminal) antibodies at 1/15,000 dilution. An additional PSII complex mentioned in the text is indicated: CV (**, PSII monomer).

Protection of PSII and PSI complexes was further analyzed by immunoblot detection in Blue-Native gels of complexes that incorporate either the D1, or the PsaB protein, respectively (Figure 4). We detected not only the dimeric PSII complexes and PSII super-complexes, but also the partially assembled CV (PSII monomer) and CVII (CP43-less PSII monomer) complexes ([17], see Figure 4A, top). Using anti-PsaB antibodies, the monomeric PSI complex and the PSI-LHCI super-complex were also detected (Figure 4A, bottom). Under control conditions the complexes containing PSII or PSI were similar in samples from the T and NT seedlings. The H_2_O_2_ treatments destroyed most PSII and PSI complexes of the NT seedlings, and only some monomeric PSI, and CVII complexes of PSII persisted (Figure 4A). In contrast in the T seedlings, fully assembled PSII complexes endured the H_2_O_2_ treatment. In addition, partially assembled D1-containing complexes -in particular the CVII complex- accumulated to higher levels in stressed T than in NT seedlings (Figure 4A, top, H_2_O_2_ samples). The PSI-LHCI super-complex also tolerated the H_2_O_2_ treatment in the T seedlings (Figure 4A, bottom). Protection of the D1-containing PSII complexes and super-complexes was confirmed in the T seedlings after treatments of severe dehydration. These complexes persisted in the T and NT seedlings immediately after dehydration [Figure 4B, samples DT (0 h)]. However, 16 h following rehydration after the dehydration treatment, the PSII complexes of T seedlings resisted the treatment better than the NT seedlings. In this case, the CV complex is the partially disassembled D1-containing complex that accumulated to higher level in the T seedlings after the stress treatment [Figure 4B, samples DT (16 h)]. Thus, we confirmed structural protection of the two photosystems.

### Protection of the D1 protein from damage and shielding of PSII repair following oxidative stress and dehydration in the 35S:A9 seedlings. Protection of PSI

**Figure 5:**
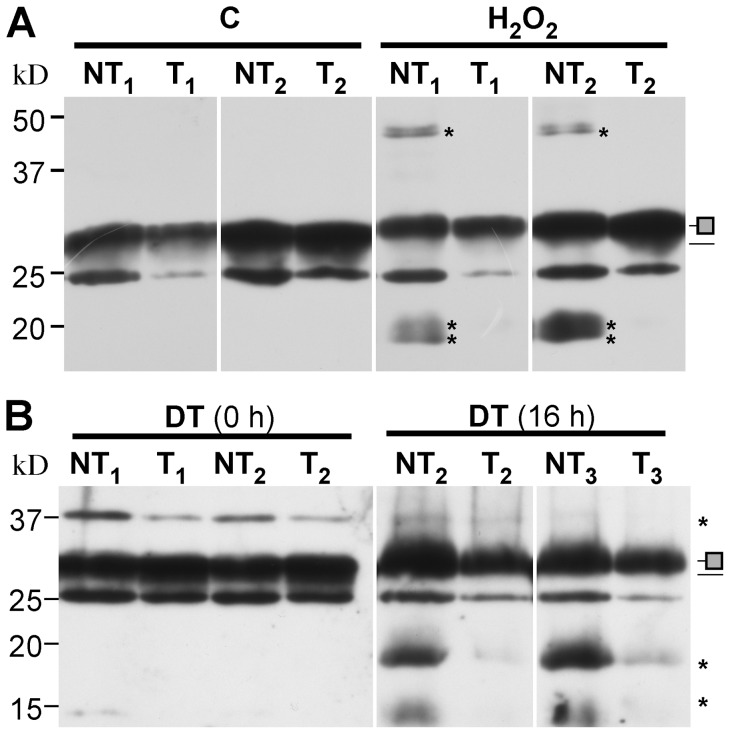
The D1 protein of PSII is protected from damage caused by oxidative stress and severe dehydration. (**A**) Damage by the H_2_O_2_ treatments (H_2_O_2_) compared to control conditions (C). (**B**) Protection from damage by the dehydration treatments (DT). The DT samples were analyzed immediately after the dehydration treatment (0 h), and following rehydration for 16 h (16 h). Thylakoid protein samples from the indicated transgenic lines (T) were compared with sibling non-transgenic (NT) lines. Protein amounts loaded per lane in order to obtain similar initial amounts of the D1 protein: NT_1_ (40 μg), T_1_ (10 μg), NT_2_ (20 μg), T_2_ (10 μg), NT_3_ (20 μg), and T_3_ (10 μg). Immunoblot detection was performed with anti-D1 (DE-loop) antibodies at 1/2,000 (A) or 1/6,000 dilution (B). To the right of each panel, a thin line marks the position of the major D1 band; slightly above, a shaded square marks the retarded mobility D1 band observed after the stress treatments. Asterisks above and below the major D1 bands respectively indicate the cross-linked and degraded D1 protein bands mentioned in the text. Molecular mass markers (kD) are indicated to the left.

We analyzed if the D1 protein of PSII is protected under the drastic H_2_O_2_ and dehydration stress conditions used above to study the 35S:A9 seedlings. The preliminary characterization of the seedlings revealed that in our experimental control conditions the T seedlings had higher content of D1 protein than the sibling NT seedlings (Figure S2). In the following analyses, the NT and T protein samples were compared using immunoblots that were normalized attempting to show equal initial (control) levels of the D1 protein signals. To this end, higher amounts of total thylakoid-membrane protein were applied for NT samples. In that way the initial amount of detected D1 protein was similar for each pair of the NT and T samples. This facilitated visual comparison of the abundance of the different D1 forms detected in immunoblot analyses after the stress treatments: e.g., the intact protein, D1-protein adducts, and D1-degradation products. In Figure 5A we show representative results of samples from two pairs of sibling T and NT lines analyzed immediately after 200 mM H_2_O_2_ treatments performed in the dark. Consistent with the requirement of light for the degradation of the D1 protein, the initial accumulation level of the D1 protein detected in the NT and T samples before treatment did not substantially decrease during the incubation with H_2_O_2_ in darkness. A slight retardation in the mobility of the major D1 band was observed for both the NT and T samples after H_2_O_2_ treatments. However, only the NT samples showed bands that migrated above (≈44.4 kD) and below (≈20 and 19 kD) the major D1 band. These bands, which accumulated at low levels in the dark, most likely represent cross-linked adducts of D1 with closely located thylakoid proteins, and initial degradation products of the D1 protein, respectively. The observation for the treated NT samples would thus be similar to previous results describing damage of the D1 protein after *in vitro* exposure to H_2_O_2_ in the dark [20]. In contrast, D1 appears to be protected from oxidative damage in the treated T samples, as no cross-linked D1-bands or degradation products were detected (Figure 5A). In Figure 5B we depict the results of a similar analysis of samples from NT/T lines after severe dehydration (DT) treatment. There, immediately after DT no D1-degradation products were detected; however, the NT samples clearly show higher accumulation of a different D1-crosslinked band of ≈36 kD (Figure 5B, samples 0 h). Only following rehydration for 16 h after DT additional changes and differences between the NT and T samples were observed (Figure 5B, samples 16 h). These differences were similar to those found as consequences of the H_2_O_2_ treatments (compare Figures 5A and 5B). The results summarized in Figure 5 clearly show that the D1 protein of the PSII reaction center of the 35S:A9 plants was protected against the oxidative stress that occurs as a result of the severe dehydration and H_2_O_2_ treatments *in vivo*.

**Figure 6:**
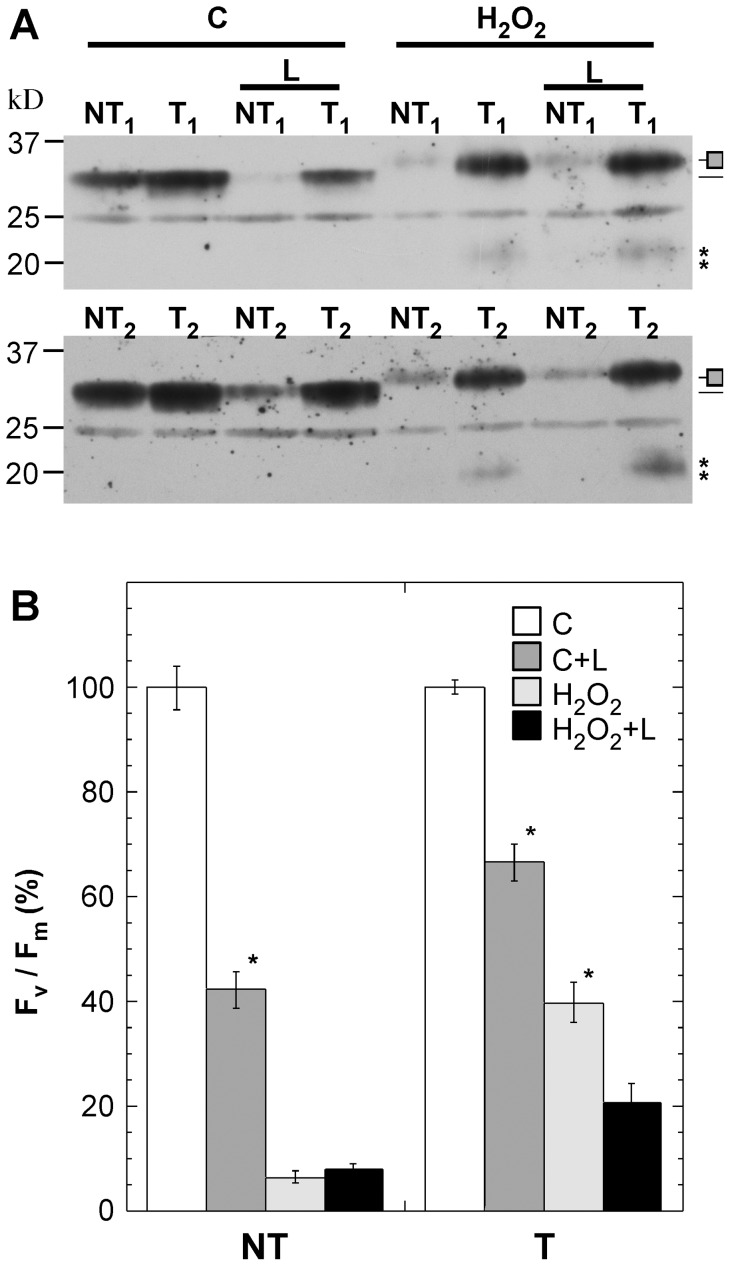
Delayed degradation of the D1 protein and protection of the D1 protein synthesis in the 35S:A9 seedlings. (**A**) Accumulation of the D1 protein under control growth conditions (C) and after treatments with H_2_O_2_ (H_2_O_2_). In each case the seedlings were exposed to 6 h normal light conditions in absence or in presence of 1mM lincomycin (L). Sample labels and rest of symbols as in Figure 5A. Protein amounts loaded per lane as in Figure 5. (**B**) Comparison of maximum quantum yield (F_v_/F_m_) of PSII between 35S:A9 seedlings (T) and sibling NT seedlings in the experimental conditions used in (A). We show average values from a representative experiment performed with three different T/NT line pairs (n = 18, for all conditions). Asterisks indicate the significant statistical differences found between the F_v_/F_m_ values obtained in absence and presence of lincomycin: for control NT seedlings (F = 111.6, P = 0.0001); for control T seedlings, (F = 59.21, P = 0.0001); and for stressed T seedlings (F = 37.75, P = 0.0001).

The synthesis of plastid-encoded components of PSII -as the D1 protein- has been shown to be rapidly inactivated by different forms of stress that cause oxidative damage (see for example [21] and references therein). Because the T seedlings survive DT [2] and drastic H_2_O_2_ treatments (Figure 3A) that involve ROS-induced damage, protection and/or reversible inactivation of PSII repair must also occur in the 35S:A9 plants. To test this, we analyzed the effects of lincomycin, a specific inhibitor of plastidial protein synthesis. Following 24 h treatments of seedlings in the dark in the absence or presence of 200 mM H_2_O_2_, the control and the stressed seedlings were transferred to normal illumination conditions for 6 h with or without the addition of lincomycin. In Figure 6A we depict the effect of lincomycin on D1 protein accumulation (the initial NT/T D1 protein levels were normalized as in Figure 5). Apparently under control conditions, normal illumination in the presence of the inhibitor can damage the D1 protein. Thus, the D1 protein of control NT seedlings showed a net degradation only in the presence of lincomycin (Figure 6A, C samples). This damage is clearly much lower in the control T seedlings, which showed higher levels of D1 protein in the presence of lincomycin. Therefore, we infer that protection of the D1 protein in T seedlings delays D1 degradation compared to NT seedlings even under control conditions. After H_2_O_2_ stress conditions protection and delayed degradation of the D1 protein in the T seedlings was even more evident; furthermore, it occurred to the same extent both in the absence and presence of lincomycin (Figure 6A, H_2_O_2_ samples). The accumulation level of the D1 protein in the H_2_O_2_-treated T seedlings was very similar to that of control T seedlings. In contrast, we observed substantial degradation of the D1 protein in the NT seedlings after exposure to H_2_O_2_ stress conditions, as demonstrated by a drastic reduction of D1 accumulation levels. That reduction also occurred both with and without the lincomycin treatment. We thus confirmed protection and delayed degradation of D1 in the stressed T seedlings. That lincomycin treatment did not have an additional effect on D1 accumulation after the stress treatments would fit the expected, H_2_O_2_-induced, inhibition of the protein synthesis [21] that is involved in replacement of the damaged D1 protein.

The exposure to H_2_O_2_ may inhibit the synthesis of the D1 protein in the NT seedlings to a similar extent as with the lincomycin treatment. To support this notion, the seedlings used for the protein analyses of Figure 6A were characterized in parallel for the indirect effects of lincomycin on the maximal quantum efficiency of PSII (Figure 6B). Lincomycin treatment significantly reduced the F_v_/F_m_ values of control NT seedlings, and a similar reduction, although lower, was also significant for control T seedlings (Figure 6B, compare C and C+L values). H_2_O_2_ treatment of the NT seedlings drastically decreased the F_v_/F_m_ values to very low levels. In contrast, the stressed T seedlings showed significantly higher F_v_/F_m_ values. These results confirm protection of PSII in the T seedlings, both under control and H_2_O_2_ stress conditions. Lincomycin treatment did not further decrease the marginal F_v_/F_m_ value observed for the H_2_O_2_-treated NT seedlings. However, for the H_2_O_2_-treated T seedlings a significant decrease of F_v_/F_m_ was observed upon lincomycin treatment (Figure 6B, compare H_2_O_2_ and H_2_O_2_+L values). This result would indicate both the protection from oxidative stress and the partial recovery of the plastidial protein synthesis involved in the repair of PSII in the 35S:A9 plants. In contrast, the PSII and the plastidial protein synthesis of the sibling NT plants would be irreversibly damaged, which contributes to the observed eventual death of these seedlings (see Figure 3A). In Figure S3, we present similar results using 35S:A9 plants subjected to DT. An indication of the recovery of plastidial protein synthesis was also observed only for the T plants. Sixteen hours after DT lincomycin significantly further reduced the F_v_/F_m_ values (Figure S3, difference indicated by the asterisk, F = 103.6, P = 0.0001). Thus, the recovery after DT appears to be delayed in comparison to what occurs after H_2_O_2_ stress.

The effects of the H_2_O_2_ treatments on the accumulation of the PSI core protein PsaB were also analyzed. Using the same control and H_2_O_2_-treated samples as in Figure 6A (the samples without subsequent treatment with lincomycin), complete degradation of PsaB in the NT seedlings was observed. In contrast, PsaB was still detected in the H_2_O_2_-treated T seedlings (Figure S4). Protection of the PsaB protein thus further confirmed the structural protection of PSI observed in these seedlings (Figure 4A, bottom).

### The heat-acclimation response does not confer resistance to the drastic oxidative stress conditions tolerated by the 35S:A9 seedlings

**Figure 7:**
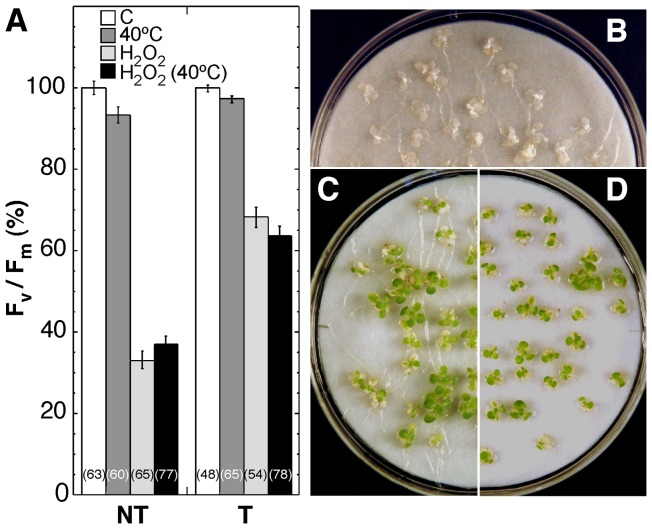
Heat acclimation does not protect PSII from damage caused by the drastic oxidative stress conditions. (**A**) Maximum quantum yield (F_v_/F_m_) of PSII for 35S:A9 seedlings (T) compared to sibling NT seedlings. The effects of standard H_2_O_2_ treatments were analyzed as in Figure 3B (compare the C and H_2_O_2_ samples), and using seedlings that were first heat-acclimated during 3 h at 40°C. The corresponding control and H_2_O_2_-treated samples are labeled 40°C and H_2_O_2_ (40°C), respectively. The average values for three to four experiments performed with three different T/NT line pairs are shown. Numbers in brackets indicate the different F_v_/F_m_ determinations performed in each case. Heat-acclimation did not induce significant differences in F_v_/F_m_ after H_2_O_2_ treatments in NT (F = 1.94, P = 0.16) or T (F = 3.32, P = 0.07) seedlings. (**B**) The heat-acclimated NT seedlings did not survive the H_2_O_2_ treatments. (**C**) Survival of non-acclimated T seedlings. (**D**) Survival of heat-acclimated T seedlings.

The HSFA9 program includes the seed expression of genes that encode HSPs of different kinds. Some of these HSPs also accumulated in seedlings in response to the sub-lethal heat stress acclimation conditions (3 h at 40°C) used to induce acquired thermo-tolerance [2]. It was previously shown [2] that heat-acclimated NT seedlings withstand treatments of 2.5 h at 50°C but are killed by the severe dehydration conditions (the DT2 treatments). In contrast, sibling T (35S:A9) seedlings withstand DT2 but are killed by the 50°C treatment unless previously heat-acclimated [2]. Here it was investigated if the same observation applies to resistance to the unusually high levels of oxidative stress imposed by the harsh H_2_O_2_ treatments used in this study (Figure 7). The effects of the H2O2 treatments as in Figure 3B (compare the C and H2O2 samples) were analyzed using also seedlings that were first heat-acclimated during 3 h at 40°C [2]. Heat-acclimation did not prevent the decrease of F_v_/F_m_ in the stressed NT seedlings; neither did it enhance the protection observed in the stressed T seedlings (Figure 7A). The heat-acclimated NT seedlings did not survive treatments for 24 h with 200 mM H2O2 in the dark (Figure 7B). In addition, the heat-acclimated T seedlings survived these treatments to the same extent as observed with non-acclimated T seedlings (Figures 7C and 7D). The genetic program (s) activated in vegetative organs by heat are thus insufficient for conferring resistance to the oxidative stress conditions used in the present study. Such conditions, however, are tolerated by the 35S:A9 seedlings, which overexpress the seed HSFA9 program.

## Discussion

The ectopic activation of the HSFA9 program in vegetative tissues of transgenic tobacco led to phenotypes of tolerance to drastic dehydration [2]. Here, we found that these phenotypes include tolerance to very severe oxidative stress and protection of the photosynthetic apparatus (Figures 2, 3, 4, 5, 6, 7). The HSFA9 program is seed-specific and thus our results suggest that such program is involved in the protection of seed non-photosynthetic plastids and proplastids, since these and other organelles must survive developmental desiccation.

The HSFA9 program conferred protection against severe dehydration and oxidative damage of photosynthetic membranes, PSII and core components of PSII as the D1 protein, as well as protection of PSI and its core protein PsaB. The protection appears to occur at different structural levels. We observed that both the D1 protein and its plastidial synthesis were resistant to damage (Figures 5 and 6). The integrity of supra-molecular membrane-protein complexes was also preserved to some extent; for example that of super-complexes with dimeric PSII, or PSI-LHCI (Figures 2B, 3C and 4). The *de novo* synthesis required for replacement of damaged proteins in the reaction center of PSII -in particular that of the D1 protein- affects the stability of PSII complexes in thylakoids; such complexes collapse under different experimental conditions where the synthesis is blocked (reviewed in [15], [22]). This suggests that the protection of the D1 protein and its plastidial synthesis might contribute to explaining the observed higher stability of PSII-containing complexes and super-complexes in the stressed 35S:A9 plants. In fact, we get converse effects on PSII as compared to mutants where a decreased stability of the PSII complexes was observed in coincidence with lower D1 protein accumulation/stability and lower D1-synthesis rates than in the WT. This includes mutants lacking PAM68 [18], or LQY1 [23]. Both PAM68 and LQY1 are thylakoid-associated proteins that would be involved in PSII-complex assembly by enhancing the turnover and biogenesis of the D1 protein. Therefore, the protection of other thylakoid-associated proteins, such as assembly factors, might also be required to explain the complex stress resistance phenotypes of the 35S:A9 seedlings. In conclusion, protection against oxidative damage of the D1 protein and that of its repair synthesis in the plastid could in part explain the drastic resistance to dehydration of PSII complexes in the 35S:A9 seedlings. It is worth noting that in some resurrection plants, but not in normal plants, the PsaB and D1 proteins, as well as PSI and PSII complexes, appeared to be similarly protected from dark dehydration treatments similar to those used here (e.g., [12], [13]). In these homoiochlorophyllous resurrection plants, which do not dismantle photosynthetic membranes when desiccated, this protection involves limiting the structural damage to levels that are reversible (reviewed in [16]). That would be similar to what we observed in Figures 2B, 3C and 4. The severe oxidative/dehydration stress conditions used in this work would irreversibly damage not only PSII but also PSI in the NT seedlings, which do not survive the stress treatments. PSI function in vegetative tissues strictly depends on protection mechanisms that are still not well characterized. These mechanisms would suffice to cope only with normal stress levels in most plants [11]. The severe stress conditions used here would overcome the normal protection of PSI/PSII. Only the activation of seed-specific protection mechanism(s) in the 35S:A9 seedlings would allow subsistence (PSI/PSII) and repair (PSII) of the photosystems and survival of the plantlets. In resurrection plants, genetic programs that are seed-specific in normal plants operate in vegetative organs; thus, the photosynthetic machinery would be protected from drastic dehydration in a similar way as proposed here [16].

We previously discussed the dehydration levels tolerated by whole 35S:A9 seedlings and by vegetative organs, such as leaves, in comparison to seeds, resurrection plants and mosses [2]. What was achieved in these seedlings is not a “canonical” desiccation tolerance as found in resurrection plants and seeds. However, the tolerated dehydration in 35S:A9 green organs is still far beyond what has been reported in any other study of non-resurrection plants. This includes genetic attempts to enhance their native tolerance (see [2] and references therein). The results presented in Figure 7 revealed that the oxidative stress conditions used in this report are as harsh as the drastic dehydration treatments (Figure 2 and [2]). Indeed, the H_2_O_2_ treatments damaged components of PSII, and even of PSI, which is considered to be resistant at least to moderate levels of stress [11]. Resistance to either very severe dehydration, or to the drastic oxidative stress conditions used in this report, was not observed after standard heat acclimation treatments (Figure 7). These acclimation treatments [2] induce a complex genetic response that in vegetative organs suffices only for coping with moderate levels of stress. In contrast, the unusual stress tolerance reported here required the activation of the seed HSFA9 program in vegetative organs of the 35S:A9 seedlings (Figure 7). Comparisons of the patterns of HSP and sHSP accumulation indicate that a partial overlap of common target genes exists between the HSFA9 program and the programs of vegetative heat-acclimation ([2]; Figure 1). We previously reasoned that the unusual dehydration-stress resistance of the 35S:A9 seedlings is explained by expression at normal growth of seed genes (HSFA9 targets) that differ from those activated by heat acclimation [2]. The present study extends that proposal. Here, we infer that only the specific target genes of the HSFA9 program can induce resistance of the photosynthetic machinery to both the severe dehydration, and to the drastic oxidative stress conditions used in our studies. The results in the present study also point to further differences in gene expression that involve plastidial sHSPs (as presented in Figure 1). Thus, the ectopic expression of different seed HSPs, including plastidial sHSPs, correlated with the observed protection in seedlings. However, we believe that it is unlikely that any single gene activated by HaHSFA9 suffices for this protection. The anti-oxidant functions demonstrated for plastidial sHSPs of higher plants [7]–[9], [24] might in part explain the observed protection of the D1 protein from oxidative damage. Thus, the 35S-driven overexpression of Arabidopsis HSP21 generated a higher abundance of the plastidial protein in unstressed leaves than induced by heat acclimation temperatures [25]. This is similar to what we observe for the HSP21-like polypeptides induced by HaHSFA9 in Figure 1 (bottom). The overexpression of HSP21 has been reported to only confer very moderate protection from oxidative damage under combined heat and high-light stress. Only subtle differences between the F_v_/F_m_ of transgenic and NT plants were observed [24]. Therefore, the overexpression of a single plastidial sHSP would not be sufficient for explaining the complex protection to extreme stress conditions that the 35S:A9 seedlings show. The association of different sHSPs with membranes has been found to decrease their fluidity, and in some cases to enhance thylakoid stability. The latter includes results obtained for sHSPs from cyanobacteria that are similar to sHSPs CI (reviewed in [26]). In summary, seed sHSPs that include plastidial sHSPs could be in part responsible for the complex protection effects observed. Any other potential target gene of HSFA9 that is involved in the protection effects reported in the present study would also not be part of the vegetative programs of thermo-tolerance.

In conclusion, the seed HSFA9 program conferred protection of photosynthetic membranes in seedlings, from damage caused *in vivo* by very harsh dehydration and oxidation. We identified some of the protected components in the two photosystems. Protection of the photosynthetic apparatus from the extreme stress conditions used in this report would be unprecedented for normal plants. The results presented here might facilitate the future engineering of stress-tolerant photosynthetic membranes, green organs, and even whole plants.

## Materials and Methods

### Plant growth conditions and stress treatments

The tobacco (*N. tabacum* L. var. Xanthi) T and NT seedlings, aged 3–4 weeks (with two pairs of true leaves), were grown on filter paper placed on Petri dishes with solid MS medium, as described [2].

In the modified dehydration treatment (DT) protocol, 3 to 4 T seedlings were placed on a glass 76×26 mm microscope slide, and the same number of the corresponding NT seedlings on a second slide. Both slides were placed alongside within the same closed glass container with 5 g silica gel and allowed to dry in the dark for 3–4 h, essentially as described for the DT2 assays [2]. This new protocol allowed using the dehydrated seedlings for immediate measurement of PSII fluorescence as described below.

For oxidative stress treatments, the filter paper with the seedlings (c.a., 60 per dish at a time) was removed and pre-washed in the solution used for the subsequent treatment: deionized water (for controls), or H_2_O_2_ (200 mM for the standard stress treatment). The H_2_O_2_ solution was prepared by diluting in deionized water the 30% w/w (8.82 M) H_2_O_2_ stock (Sigma #H1009). The final concentration was verified by measuring A_240_ (A_240_  = 1.31 for 30 mM H_2_O_2_)_._ These solutions also included 0.1% Tween−20 to facilitate seedling penetration. After removing the excess of liquid, the seedlings were put back inside Petri dishes; the seedlings placed on 6 circles of filter paper wetted with the respective treatment solution. The Petri dishes were incubated at 20–25°C in the dark for 24 h. After the treatment, the seedlings were washed with deionized water. When the stress treatment was followed by a treatment with lincomycin, the seedlings were washed in 1 mM lincomycin and placed on filter paper soaked with 1 mM lincomycin within sealed Petri dishes; the seedlings were first left for 1 h in the dark to allow lincomycin penetration, and then put under normal light conditions (daylight lamps, ≈6,000 lux) for 6 h. For assaying survival of seedlings after the H_2_O_2_ treatment and washing with deionized water, the seedlings were placed again on wet filter paper within sealed Petri dishes. The seedlings were photographed after 1 week inside growth-cabinets with photoperiod.

### Chlorophyll fluorescence

Chlorophyll fluorescence was measured with a mini-PAM Photosynthesis Yield Analyzer (Heinz Walz, Effeltrich, Germany). Immediately after the stress (or respective control) treatments, each seedling was placed between glass microscope slides (76×26 mm, bottom, and 60×24 mm, cover); the slides were fastened using DLC-8 clips. After a subsequent dark adaptation period of 25 min, minimum fluorescence (F_o_) was determined by a weak red light. Maximum fluorescence of dark-adapted state (F_m_) was measured during a subsequent saturating light pulse (14,000 μmol m^−2^ s^−1^ for 0.8 s). The maximal quantum efficiency of PSII (F_v_/F_m_) was determined as the ratio of variable fluorescence (F_v_) to F_m_. Measurements for six different seedlings per condition (e.g., control or stress treatment) were averaged in each independent experiment. F_v_/F_m_ values after the different stress treatments are represented as percent values of the respective control F_v_/F_m_ values. The seedlings were chosen to represent different locations within the Petri dish. The number of experimental repeats is indicated in the Figure legends.

### Isolation of thylakoid membranes

Thylakoid membranes were prepared essentially as described [27]. Whole-seedling material was frozen and reduced to a fine powder using liquid N_2_ in a cooled mortar; then it was homogenized under weak ambient light in ice-cold isolation buffer (330 mM sucrose, 25 mM HEPES-KOH, pH 7.4, 10 mM MgCl_2_, and 10 mM NaF) supplemented with 1 mM Pefablock (Roche). Approximately 1 mL of buffer was used per 200 mg of seedling powder. Extracts were collected after filtration through Miracloth (Calbiochem). The filtrate was then centrifuged at 4°C for 5 min at 6,000*g*; pellets with the membranes were gently re-suspended in isolation buffer without sucrose, and washed in this buffer by centrifugation and re-suspension in the same conditions. The washed thylakoid membranes were re-suspended in Pefablock-supplemented isolation buffer (using 1 μL buffer per mg of starting frozen material), and stored at −80°C in aliquots of ≈50 μL. The protein content of the thylakoid membranes was quantified using the modification of the Lowry procedure by Markwell *et al*. [28].

### Protein analyses

The procedures for the analyses performed with seedling total protein extracts were as described [1], [2]. 1D-PAGE with thylakoid protein extracts was performed similarly, only that the 12.5% polyacrylamide gels and sample buffer contained 4 M Urea. Immunoblot procedures using PVDF membranes were essentially as described [1], [2]. The following primary antibodies -obtained from Agrisera, Sweden- were used in this study, at dilutions indicated in each case in the corresponding Figures: Arabidopsis HSP21 (AS08285); D1 protein (PsbA, anti-C-terminal and anti-DE-loop, respectively AS05084, AS10704); PsaB (AS10695).

### BN-PAGE

1D-BN-PAGE was performed as described [29] with slight modifications. Samples of washed thylakoids (100 μg total protein) were re-suspended in 10 μL 20% (w/v) glycerol, 25 mM BisTris-HCl, pH 7.0. An equal volume of re-suspension buffer containing 2% β-dodecyl-maltoside was gently mixed within a micro-pipet tip. Samples then were incubated for 30 min at 4°C, the insoluble material removed by centrifugation; the solubilized material was combined with Serva-Blue G and loaded into 0.75-mm-thick 5–13.5% acrylamide gradient gels [29]. These gels were run for 4–5 h at constant voltage (150 V) at 4–8°C. The cathode buffer was exchanged with buffer lacking dye after 2 h electrophoresis; the electrophoresis was stopped when most of the front-moving dye exited the gel. BN-Gels were then directly photographed, stained with colloidal Coomassie Brilliant Blue G-250 [30], or transferred to PVDF membranes. The immunoblot analyses after BN-PAGE differ from those previously described [1], [2] in that: 1.– Blocking with TBST was prolonged overnight to facilitate removal of the bound Serva-Blue G dye from the membrane. 2.– Hybridization with the primary antibodies was for 4 h at 25°C.

### Statistical analyses

Differences between T and NT groups of sibling seedlings were tested by analysis of variance (ANOVA). The statistical analyses were performed as described in detail [1], [2].

## Supporting Information

Figure S1. **The HSP21-like polypeptides in the 35S:A9 seedlings associate with thylakoid membranes at normal growth temperatures.** (**A**) Washing of thylakoid membranes removes contamination from stromal HSP70B. Comparison of signals obtained for total protein extracted from seedlings (Tot) and from pellets (P) of thylakoid preparations that where washed once (W1) or four times (W4). The amounts of loaded protein corresponded to an equivalent amount of chlorophyll: 2 μg in all lanes. Antibodies against the Arabidopsis HSP70B protein (Agrisera AS08348) were used at 1/8,000 dilution. (**B**) Comparison of the HSP21 and PsbP proteins in pellet (P) and soluble (S) fractions after treatments with 1 M NaCl or 0.8 M Tris-HCl, pH 8.4, for 60 min at 25°C, followed by centrifugation for 20 min at 16,000 g and 4°C. Protein amounts in each fraction were compared with the initial total amounts in thylakoid pellets that were washed four times (W4/P). These amounts correspond to 0.5 μg (sHSP-P detection), or 0.2 μg of chlorophyll (PsbP detection). Antibodies against HSP21 or PsbP (Agrisera AS06167) were both used at 1/3,000 dilution.
(TIF)

Figure S2. **Enhanced accumulation of the D1 protein in thylakoid membranes of the 35S:A9 seedlings.** The 3 pairs of T/NT sibling lines were compared. Sample amounts of thylakoid protein loaded: 0.15 μg for D1 detection in all lanes. Equal loading was verified with Ponceau S staining (Po) using a higher amount thylakoid protein from the same samples: 20 μg. Antibodies against the C-terminal region of the D1 protein were used at 1/15,000 dilution. Molecular mass markers (kD) are indicated.
(TIF)

Figure S3. **Protection of plastidial protein synthesis in the dehydrated 35S:A9 seedlings.** Maximum quantum yield (F_v_/F_m_) of PSII for 35S:A9 seedlings (T) compared to sibling NT seedlings. The effect of 1 mM lincomycin (L) was analyzed. Lincomycin was added during 16 h of rehydration under normal light conditions (R) of seedlings subjected first to dehydration treatments [DT (R)]: compare DT (R) with DT (R)+L. The F_v_/F_m_ values obtained immediately after dehydration are also indicated (DT). We show average values from three independent experiments performed with two different T/NT line pairs. Numbers in brackets indicate the total number of F_v_/F_m_ determinations in each condition.
(TIF)

Figure S4. **The PsaB protein of PSI is protected from damage caused by oxidative stress in the 35S:A9 seedlings.** The same protein samples from experiments analyzed in Figure 7A for D1 protection were used here. Immunoblot detection was performed using anti-PsaB antibodies at 1/5,000 dilution. Sample labels are described in the legend of Figure 7A.
(TIF)
