# Supplementary figures and images for: Protection of the Photosynthetic Apparatus from Extreme Dehydration and Oxidative Stress in Seedlings of Transgenic Tobacco
Source: PLoS One. 2012 Dec 5;7(12):e51443. doi: 10.1371/journal.pone.0051443 (PMC3515515; doi:10.1371/journal.pone.0051443)

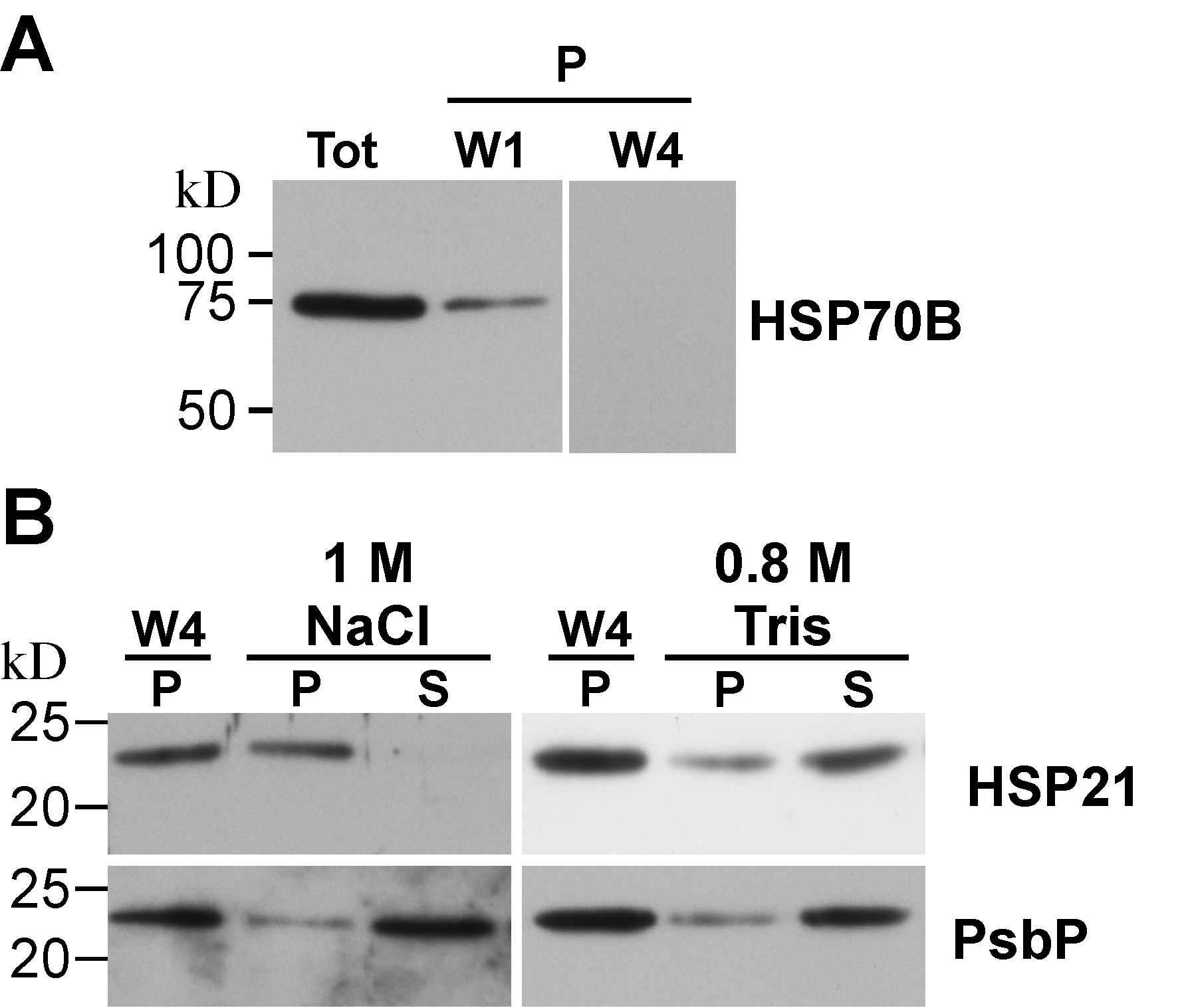

Supplement: Figure S1. — The HSP21-like polypeptides in the 35S:A9 seedlings associate with thylakoid membranes at normal growth temperatures. (A) Washing of thylakoid membranes removes contamination from stromal HSP70B. Comparison of signals obtained for total protein extracted from seedlings (Tot) and from pellets (P) of thylakoid preparations that where washed once (W1) or four times (W4). The amounts of loaded protein corresponded to an equivalent amount of chlorophyll: 2 μg in all lanes. Antibodies against the Arabidopsis HSP70B protein (Agrisera AS08348) were used at 1/8,000 dilution. (B) Comparison of the HSP21 and PsbP proteins in pellet (P) and soluble (S) fractions after treatments with 1 M NaCl or 0.8 M Tris-HCl, pH 8.4, for 60 min at 25°C, followed by centrifugation for 20 min at 16,000 g and 4°C. Protein amounts in each fraction were compared with the initial total amounts in thylakoid pellets that were washed four times (W4/P). These amounts correspond to 0.5 μg (sHSP-P detection), or 0.2 μg of chlorophyll (PsbP detection). Antibodies against HSP21 or PsbP (Agrisera AS06167) were both used at 1/3,000 dilution. (TIF) [file pone.0051443.s001.tif]

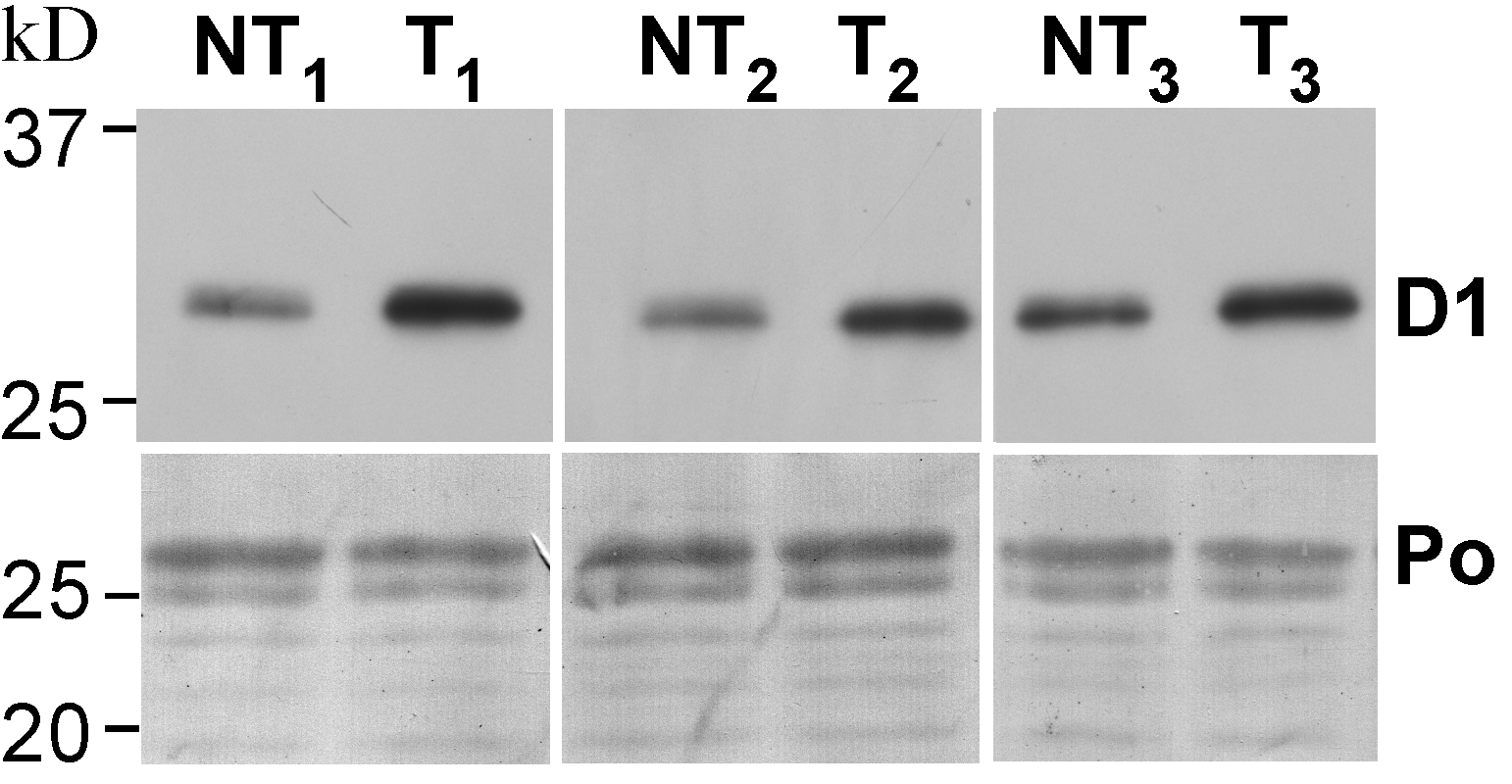

Supplement: Figure S2. — Enhanced accumulation of the D1 protein in thylakoid membranes of the 35S:A9 seedlings. The 3 pairs of T/NT sibling lines were compared. Sample amounts of thylakoid protein loaded: 0.15 μg for D1 detection in all lanes. Equal loading was verified with Ponceau S staining (Po) using a higher amount thylakoid protein from the same samples: 20 μg. Antibodies against the C-terminal region of the D1 protein were used at 1/15,000 dilution. Molecular mass markers (kD) are indicated. (TIF) [file pone.0051443.s002.tif]

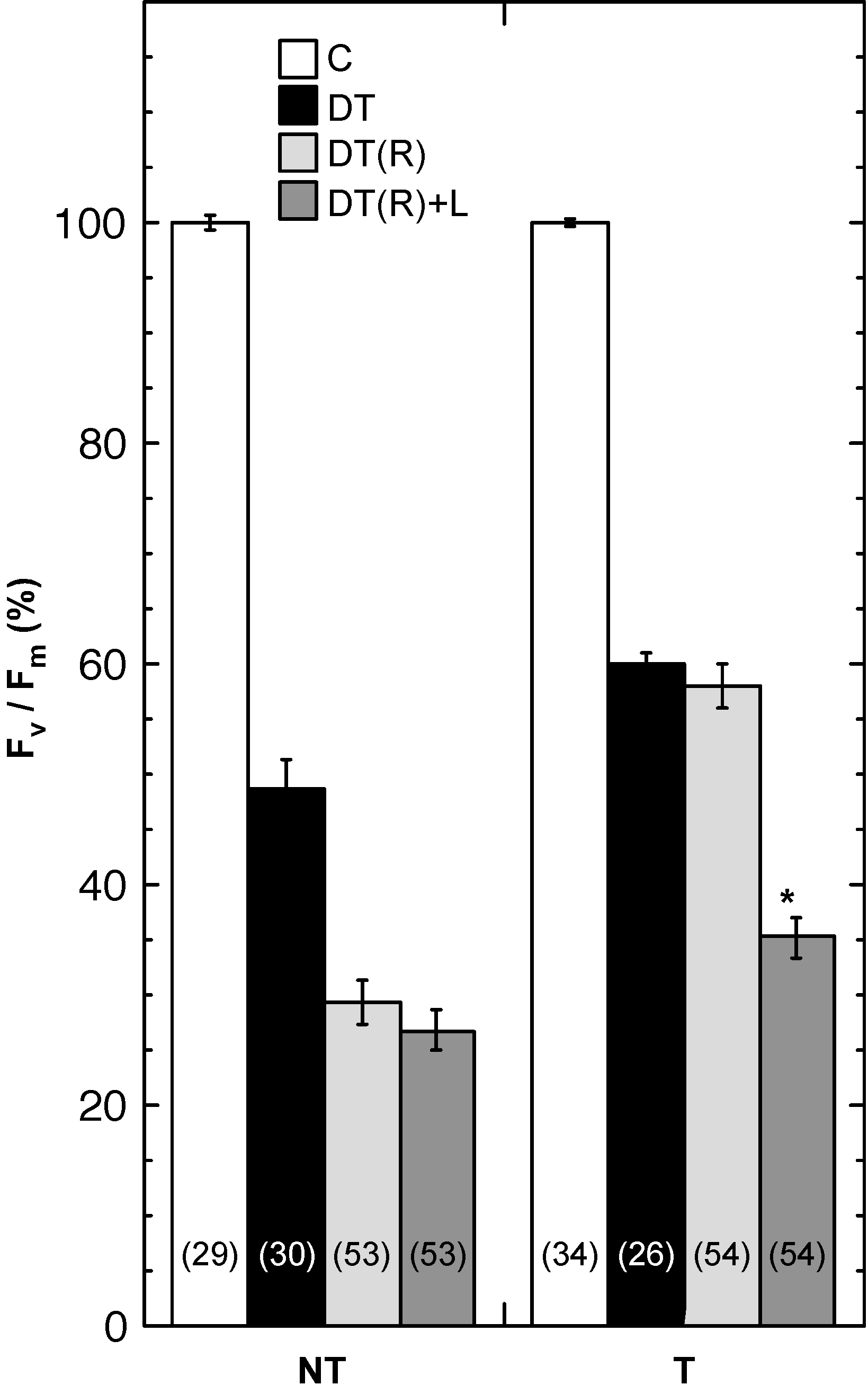

Supplement: Figure S3. — Protection of plastidial protein synthesis in the dehydrated 35S:A9 seedlings. Maximum quantum yield (Fv/Fm) of PSII for 35S:A9 seedlings (T) compared to sibling NT seedlings. The effect of 1 mM lincomycin (L) was analyzed. Lincomycin was added during 16 h of rehydration under normal light conditions (R) of seedlings subjected first to dehydration treatments [DT (R)]: compare DT (R) with DT (R)+L. The Fv/Fm values obtained immediately after dehydration are also indicated (DT). We show average values from three independent experiments performed with two different T/NT line pairs. Numbers in brackets indicate the total number of Fv/Fm determinations in each condition. (TIF) [file pone.0051443.s003.tif]

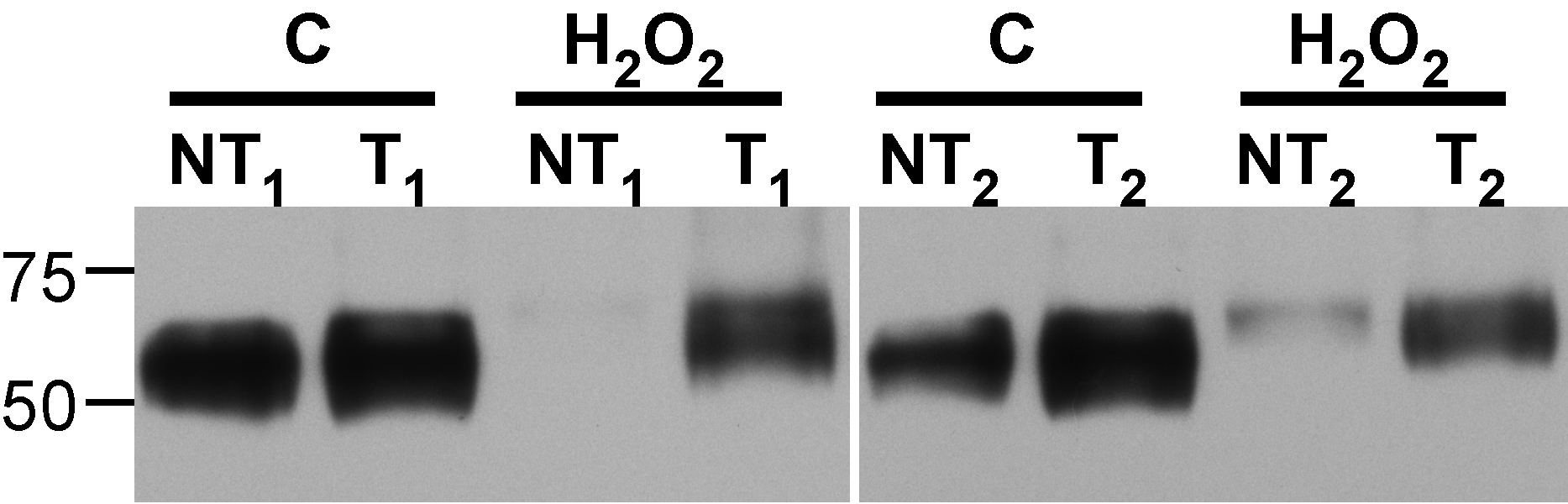

Supplement: Figure S4. — The PsaB protein of PSI is protected from damage caused by oxidative stress in the 35S:A9 seedlings. The same protein samples from experiments analyzed in Figure 7A for D1 protection were used here. Immunoblot detection was performed using anti-PsaB antibodies at 1/5,000 dilution. Sample labels are described in the legend of Figure 7A. (TIF) [file pone.0051443.s004.tif]
